# Low Normal TSH levels are Associated with Impaired BMD and Hip Geometry in the Elderly

**DOI:** 10.14336/AD.2016.0325

**Published:** 2016-12-01

**Authors:** Su Jin Lee, Kyoung Min Kim, Eun Young Lee, Mi Kyung Song, Dae Ryong Kang, Hyeon Chang Kim, Yoosik Youm, Young Mi Yun, Hyun-Young Park, Chang Oh Kim, Yumie Rhee

**Affiliations:** ^1^Department of Internal Medicine, Severance Hospital, Endocrine Research Institute, Yonsei University College of Medicine, Seoul, Korea; ^2^Department of Internal Medicine, National Health Insurance Service Ilsan Hospital, Goyang, Korea; ^3^Department of Internal Medicine, Seoul National University Bundang Hospital, Seongnam, Korea; ^4^Division of Endocrinology and Metabolism, Department of Internal Medicine, Seoul St. Mary’s Hospital, The Catholic University of Korea, Seoul, Korea; ^5^Department of Research Affairs, Biostatistics Collaboration Unit, Yonsei University College of Medicine, Seoul, Korea; ^6^Office of Biostatistics, Ajou University School of Medicine, Suwon, Korea; ^7^Department of Preventive Medicine, Yonsei University College of Medicine, Seoul, Korea; ^8^Department of Sociology, Yonsei University, Seoul, Korea; ^9^Division of Cardiovascular and Rare Diseases, Korea National Institute of Health, Osong, Korea; ^10^Division of Geriatrics, Department of Internal Medicine, Severance Hospital, Seoul, Korea

**Keywords:** TSH, euthyroidism, elderly people, bone density, bone geometry

## Abstract

Subclinical hyperthyroidism is known to be associated with the risk of fractures in elderly people. However, there are few studies assessing whether low normal thyroid-stimulating hormone (TSH) levels affect bone density and geometry. Here, we aimed to assess the influence of the TSH level on bone mineral density (BMD) and geometry in elderly euthyroid subjects. This was a cross-sectional cohort study. A total of 343 men and 674 women with euthyroidism were included and analyzed separately. The subjects were divided into tertiles based on the serum TSH level. The BMD and geometry were measured using dual-energy X-ray absorptiometry and a hip structural analysis program. Multiple regression analysis was used to compute the odds ratios of osteoporosis in the lower TSH tertile group and the association between geometry parameters and the TSH level. We found that the femoral neck and total hip BMDs were lower in the lower TSH tertile group. In women, the cross-sectional area and cortical thickness of the femur were negatively associated with the TSH level in all three regions (the narrow neck, intertrochanter, and femoral shaft); however, in men, these geometry parameters were significantly associated with the TSH level only in the intertrochanter region. The buckling ratio, a bone geometry parameter representing cortical instability, was significantly higher in the lower TSH tertile group in all three regions in women, but not in men. Our results indicated that lower TSH levels in the euthyroid range are related to lower BMD and weaker femoral structure in elderly women.

Maintaining normal thyroid function is important for achieving appropriate bone development and peak bone mass in young age, as well as for regulating the rate of bone turnover in adults [1]. It is well known that bone mineralization is reduced and that the rate of bone turnover is increased in thyrotoxicosis [1-3], leading to an increased risk of hip fractures [4, 5]. Bauer et al. showed an association between low thyroid-stimulating hormone (TSH) levels and hip fractures in subclinical hyperthyroidism [6] and a recent meta-analysis [7] revealed increased fracture risk in cases of subclinical hyperthyroidism. Moreover, Murphy et al. reported that higher free thyroxine (fT4) levels were associated with reduced hip bone mineral density (BMD), even in euthyroid postmenopausal women [8]. Leader et al. also revealed that the risk of hip fractures was significantly higher in euthyroid women over the age of 65 years with TSH levels in the lower normal range [9].

As cortical bone loss becomes more prominent with age, integrated analysis of not only bone mass but also the geometry in the femur in this population is important. In addition, as thyroid hormones have a greater effect on cortical than trabecular bone [2, 10, 11], it may be helpful to evaluate the association between the cortical bone geometry parameters of the proximal hip and thyroid hormone levels.

Dual-energy X-ray absorptiometry (DXA) is a widely used tool for measuring conventional BMD. Although DXA cannot analyze the trabecular and cortical compartments separately, various cortical geometry parameters such as the estimated average cortical thickness (CoTh) and index of strength in bending, which can provide information on the biomechanical status, can be estimated by using a hip structural analysis (HSA) program [12].

Few studies have analyzed the impact of TSH on hip geometry parameters in subjects with normal TSH levels. Accordingly, we hypothesized that normal TSH levels in the lower range could negatively affect not only the BMD, but also femoral geometry parameters, thereby leading to an increased risk of hip fractures. In this report, in order to estimate bone geometry parameters of the proximal femur based on TSH levels, we analyzed data from the Korean Urban Rural Elderly (KURE) study [13], a cohort study of the Korean elderly population.

## MATERIALS AND METHODS

### Study subjects

**Figure 1.**
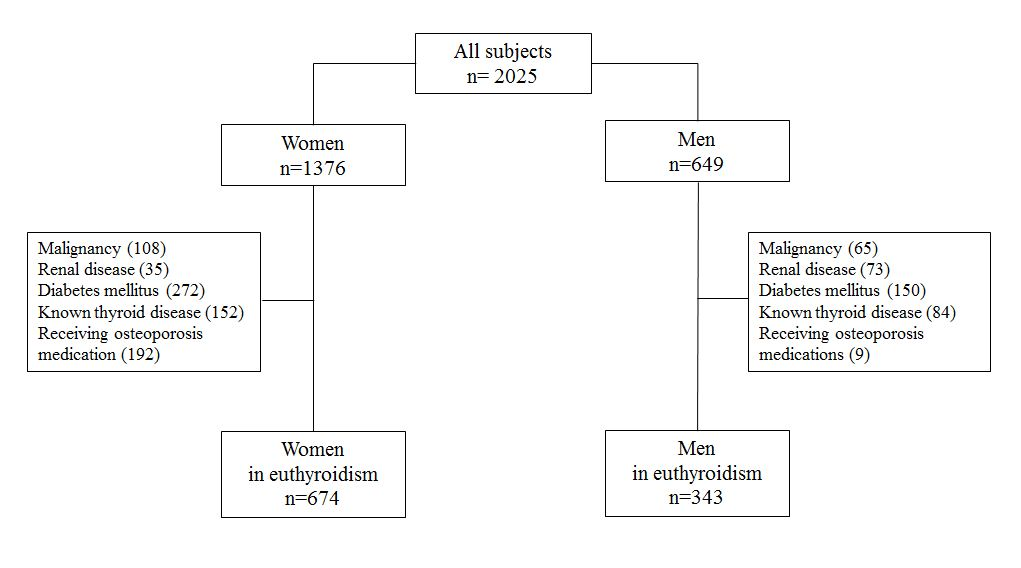
The flow chart of the study subjects

Between May 2012 and August 2013, a total of 2025 subjects were enrolled in the KURE study. The KURE study is a community-based cohort study involving community-dwelling elderly persons aged 65 years and older [13]. All subjects underwent a full health examination, including blood analysis, BMD analysis, and fracture risk assessment. We excluded all subjects with malignancies (n = 173), renal disease (n = 108), diabetes mellitus (n = 422), known hyperthyroidism or suspected hypothyroidism treated by medications (n = 64),with hormone levels beyond the normal range of fT4 (0.89-1.76 ng/dL) and TSH (0.35-5.5 µIU/mL), presenting with subclinical hyperthyroidism or hypothyroidism at the time of the first visit (n = 147), and on any medications that could affect bone metabolism such as corticosteroids, bisphosphonates, and selective estrogen receptor modulators (n = 201) (Fig. 1). All subjects provided written informed consent, and the Institutional Review Board of Yonsei University Health System approved this study (IRB No. 4-2012-0172).

### Biochemical parameters

Blood samples were collected in the morning after an overnight fast for at least 8 hours. Serum T3, fT4, and TSH levels were measured by chemiluminescence immunoassay using the ADVIA Centaur XP Immunoassay System (Siemens, Washington, DC, USA). The intra- and inter-assay coefficients of variation (CV) were < 1.5% and < 1.1% for T3, < 2.2% and < 4.0% for fT4, and < 2.4% and < 0.9% for TSH, respectively. Further, 25-hydroxyvitamin D (25(OH)D) was measured by chemiluminescence immunoassay using the Liaison platform (Diasorin, Germany) (intra- and inter-assay CV, < 4.2% and < 7.7%, respectively). Intact parathyroid hormone (PTH) and osteocalcin levels were measured by electrochemiluminescence immunoassay using the E-170 module (Roche Diagnostics, Indianapolis, IN, USA). The intra- and inter-assay CVs were < 1.2% and < 2.5% for PTH, and < 0.5% and < 1.4% for osteocalcin, respectively.

### BMD, geometry parameters, and fracture risk assessment

DXA (Hologic Discovery W, Hologic Inc., Bedford, MA, USA) was used for measuring the BMD of the lumbar spine and total hip. Vertebral fractures were evaluated by a DXA-based lateral vertebral assessment tool, an established semi-quantitative visual scoring system [14]. Vertebral fractures were defined as an estimated 25-40% reduction (grade 2) or a greater than 40% reduction (grade 3) in the height of any part of the vertebral body. Hip fractures and non-hip non-vertebral fractures were evaluated according to the questionnaire. Non-hip non-vertebral fractures include wrist, elbow, ankle, phalanx, rib, clavicle, knee, malleolus, and other fractures. Bone geometry and strength parameters were further analyzed using the HSA program included in the APEX software (Hologic Inc., Bedford, MA, USA). The HSA program automatically sets the region of interest (ROI) as the narrow neck (NN; across the narrowest diameter of the femoral neck), intertrochanter (IT; along the bisector of the neck-shaft angle), and femoral shaft (FS; 2 cm distal to the midpoint of the lesser trochanter) [12, 15]. For geometry analysis in HSA, the NN and FS regions were modeled as circular annuli with 60% and 100% cortical bone, respectively. The IT region was modeled as an elliptical annulus and was assumed to comprise 70% cortical bone. We analyzed five geometry parameters; the CoTh, cross-sectional area (CSA: equivalent to the amount of cortical-equivalent bone surface area in the cross-section after excluding all trabecular and soft tissue spaces [16]), buckling ratio (BR: index of cortical instability calculated by the ratio of the maximal diameter to the estimated average CoTh), cross-sectional moment of inertia (CSMI: resistance to bending forces in a cross section, and section modulus (Z: index of bending strength) for the NN, IT, and FS were all calculated. Higher values of all parameters, except for BR, represent higher femoral strength. We also calculated the 10-year fracture risk by using the World Health Organization’s fracture risk assessment tool (FRAX) for Koreans, with options for including the femoral neck areal BMD (g/cm^2^), as measured by DXA.

### Statistical analysis

To evaluate the differences according to TSH levels in the normal range, the subjects were divided into tertiles based on the TSH levels. The data for men and women were analyzed separately. Descriptive data are presented as the mean ± standard deviation (SD). Differences in the clinical characteristics of the subjects were analyzed using one-way analysis of variance (ANOVA) with Bonferroni post-hoc analysis for continuous variables and the χ^2^ test for categorical variables. Fisher exact test was used for evaluating differences in the prevalence of smoking. Multiple logistic regression analyses were used to evaluate the odds ratios (ORs) of osteoporosis with a T-score less than -2.5 of any part of the lumbar spine, femoral neck, or total hip in the lower and middle tertiles of TSH levels compared to the upper tertile of TSH levels. Multiple linear regression analysis was performed to assess the association of the TSH tertiles and bone geometry parameters. In the multivariate analysis, age, body mass index (BMI), free thyroxine, 25(OH)D, PTH, smoking, drinking, and the period of menopause (only for women) were adjusted as covariates. The ß coefficients were analyzed by setting the upper TSH group as the reference group. Dependent variables included the femoral geometry parameters for the NN, IT, and FS. *P* values < 0.05 were considered statistically significant. All statistical analyses were performed using SAS (version 9.2; SAS Institute Inc., Cary, NC, USA).

## RESULTS

### Clinical characteristics of the subjects

In the final analysis, euthyroid subjects, including 674 women (66.3%) and 343 men (32.7%), were included. To evaluate the relationship between bone parameters and TSH levels in subjects with normal thyroid function, the study population was stratified according to the tertiles of TSH level (lower, middle, upper TSH: TSH1 vs. TSH2 vs. TSH3, respectively). The characteristics of the subjects are shown in Table 1. In women, the fT4 levels were significantly different among the three groups according the tertiles of TSH level; however, no difference was observed in men. The mean osteocalcin level, which is one of the bone turnover markers, was the highest in the lower tertile group (TSH1) in both men and women; however, no statistically significant difference was noted between any groups. In our data, the prevalences of vertebral fractures and hip fractures were 23% and 1.48% in women, and 28% and 2.04% in men, respectively. Non-hip non-vertebral fractures were 25.67% in women and 20.12% in men. All previous fractures were not statistically different among the TSH tertile groups.

**Table 1:** Clinical characteristics

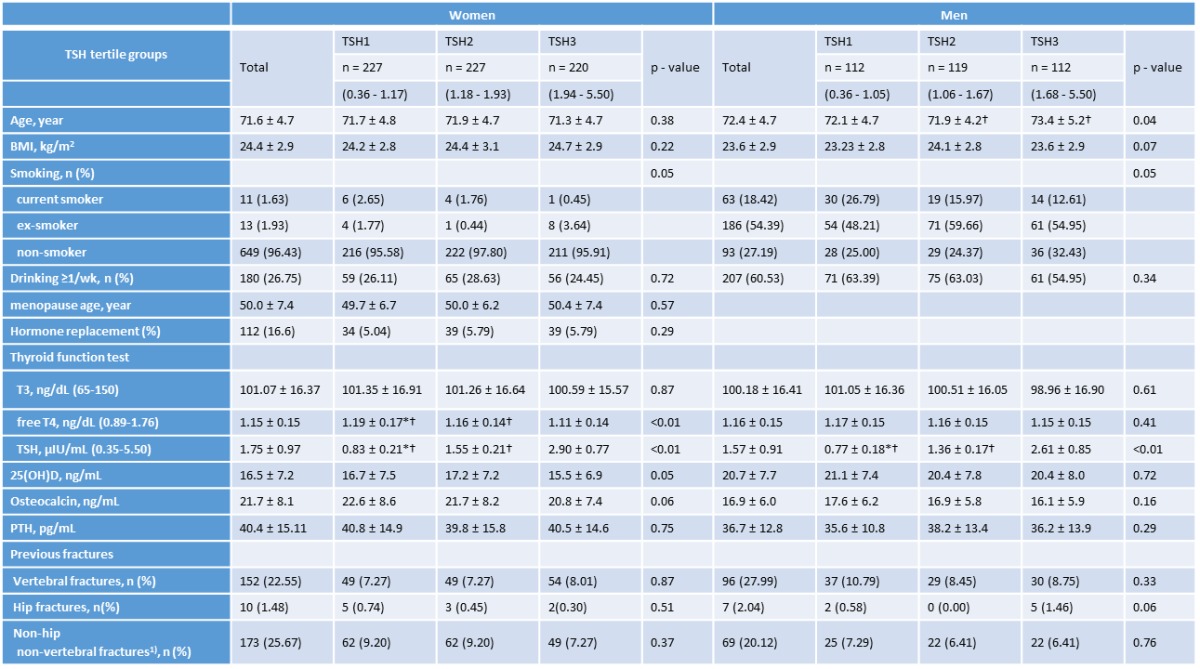

Values presented as mean ± standard deviation (SD), TSH1: lower tertile, TSH2: middle tertile, TSH3: upper tertile, BMI: body mass index, T3, triiodothyronine, T4: thyroxine, 25(OH)D: 25-hydroxyvitamin D, PTH: parathyroid hormone ^1)^ non-hip non-vertebral fractures include wrist, elbow, ankle, phalanx, rib, clavicle, knee, malleolus, and other fractures. *P* - value was calculated by ANOVA. Bonferroni’s post hoc analysis: * < 0.05 v TSH2, † < 0.05 vs TSH3

### The lower TSH tertile showed the lowest BMD and highest FRAX score

The femoral neck and total hip BMD were significantly lower in women with lower normal TSH levels (p < 0.01, and p < 0.01, respectively). While only tendency was shown in the lumbar spine (*p* for trend = 0.051) (Table 2). In addition, the FRAX 10-year risk scores of major osteoporotic fracture and hip fracture were significantly higher in the lower TSH tertile in women compared to the upper tertile (Table 2). After adjusting for age, BMI, free thyroxine, 25(OH)D, PTH, smoking, drinking, and the period of menopause, women in the lower TSH tertile had a significantly greater risk of osteoporosis compared with the upper TSH tertile group (OR 1.86, 95% confidence interval [CI] 1.22-2.83, *p* < 0.01). There was no significant difference in the incidence of osteoporosis in the lower TSH tertile of men (OR 1.30, 95% CI 0.86-1.97, *p* = 0.35) (Table 3).

### Deteriorated femoral bone geometry in the lower TSH tertile

**Figure 2.**
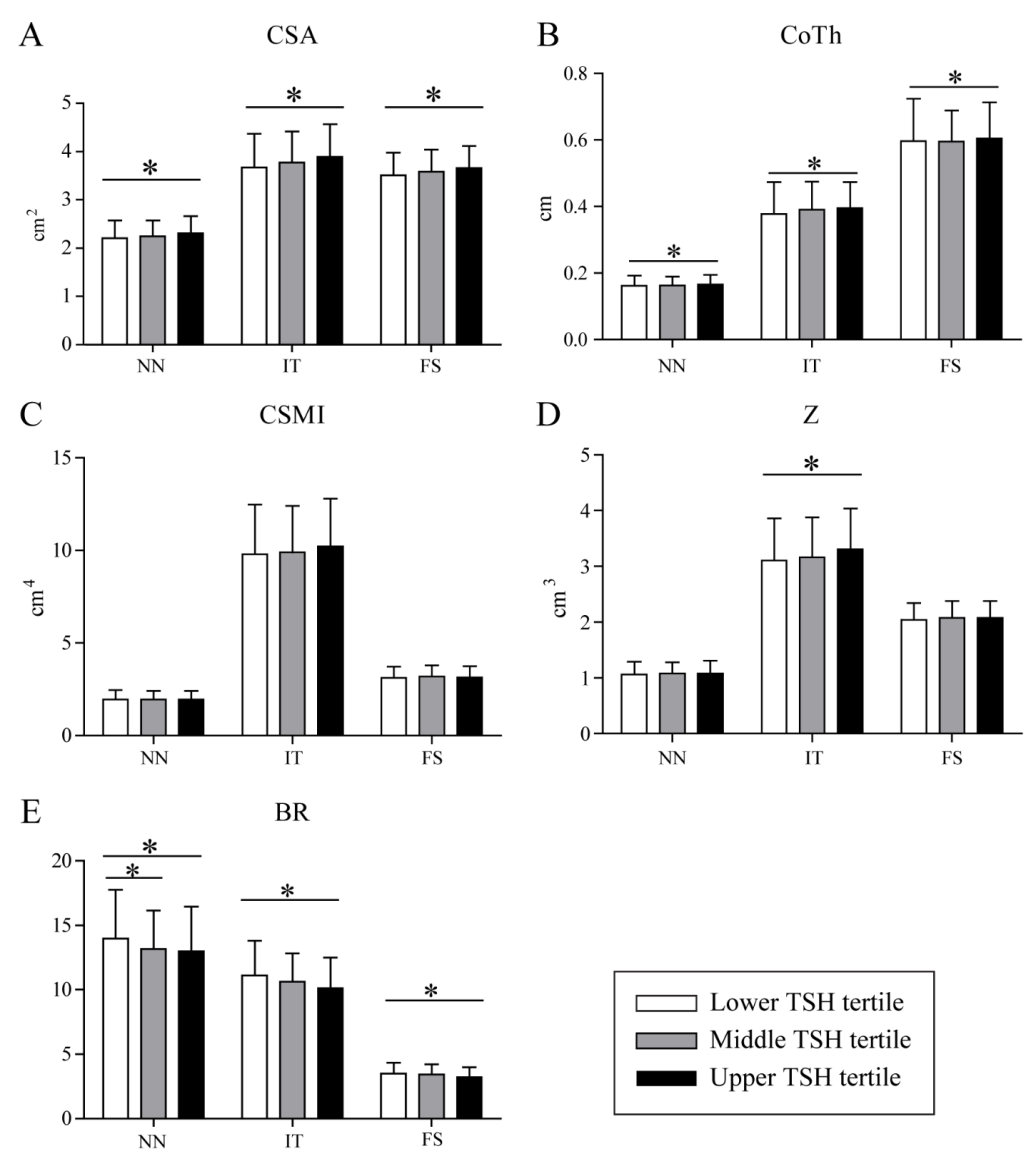
**Bone geometry parameters of the three regions according to the TSH tertiles in women**. CSA (**A**) and CoTh (**B**) in the NN, IT, and FS were significantly lower in the lower TSH tertile than in the upper TSH tertile. CSMI (**C**) and Z (**D**) were not associated with the TSH tertiles, except for Z in IT. BR (E), which indicates cortical instability, showed higher values in the lower TSH tertile. TSH: thyroid-stimulating hormone, CSA: cross-sectional area, CoTh: cortical thickness, CSMI: cross-sectional moment of inertia, Z: section modulus, NN: narrow neck, IT: intertrochanter, FS: femur shaft. Bars indicate the standard deviations. *p < 0.05 by ANOVA

Fig. 2 presents the differences in femoral bone geometry according to the TSH tertiles in the NN, IT, and FS in women. The CSA and CoTh in all three regions were significantly lower in the lower TSH tertile compared with in the upper TSH tertile (-5.0% and -7.1% in NN; -6.0% and -6.7% in IT; and -4.3% and -7.1% in FS, respectively; *p* < 0.01 for all), as was Z. Further, the BR values in all three regions were significantly higher in the lower TSH tertile as compared with those in the upper TSH tertile (7.7% in NN; 9.7% in IT; and 9.0% in FS; *p* < 0.01 for all). In men, none of the bone geometry parameters differed significantly across the TSH tertiles for all three regions (Supplemental table 1).

**Table 2:** BMD and fracture risk assessment according to the tertiles of TSH

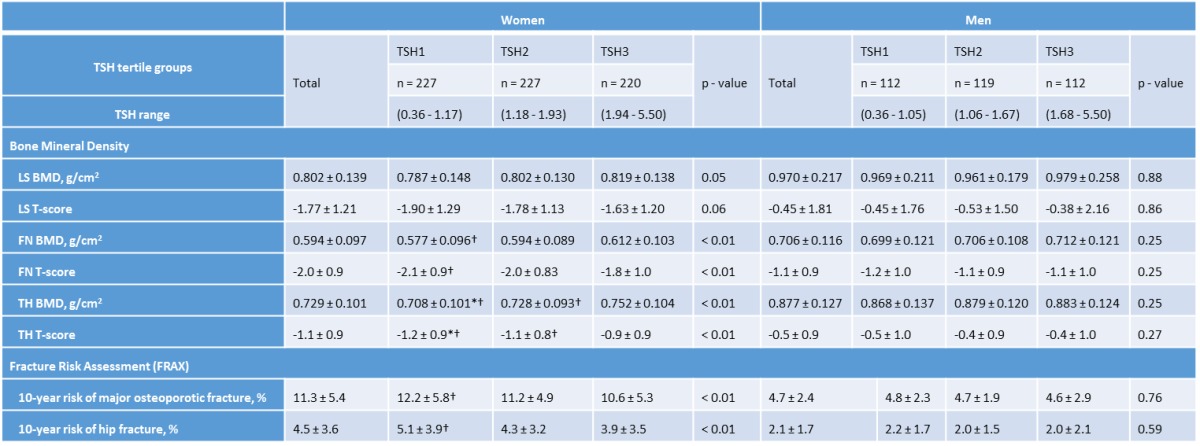

Values are presented as the mean ± standard deviation. BMD: bone mineral density, TSH: thyroid-stimulating hormone, TSH1: lower tertile, TSH2: middle tertile, TSH3: upper tertile, LS: lumbar spine, FN: femoral neck, TH: total hip. Bonferroni’s post hoc analysis: *p < 0.05 vs. TSH2, †*p* < 0.05 vs. TSH3. *P*-value for trend test adjusted by age, body mass index, free thyroxine, 25-hydroxyvitamin D, parathyroid hormone, smoking, and drinking, the period of menopause (only for women).

Next, to evaluate the association between the TSH tertiles and femoral bone geometry parameters, we performed multiple linear regression analysis after adjusting for confounding covariates. We set the upper TSH tertile (TSH3 group) as the reference group. Among women, subjects in the lower TSH tertile showed the lowest CSA (NN: ß = -0.096, *p* = 0.02; IT: ß = -0.113, *p* < 0.01; FS: ß = -0.096, *p* = 0.02) and lowest CoTh (NN: ß = -0.130, *p* < 0.01; IT: ß = -0.140, *p* < 0.01; FS: ß = -0.138, *p* < 0.01) in all three regions (Table 4). However, CSMI and Z were not associated with the TSH tertiles, except for Z in IT (ß = -0.143, *p* = 0.03) (Fig. 2). Further, BR, which is higher as the cortical instability increases, was the highest in the lower TSH tertile, indicating that the cortical bone was thinner and more unstable in the lower TSH tertile than in the upper TSH tertile in all three regions (NN: ß = 0.111, *p* = 0.01; IT: ß = 0.160, *p* < 0.01; FS: ß = 0.142, *p* < 0.01). In men, all five geometry parameters (CSA, CSMI, Z, CoTh, and BR) showed a significant association across the TSH tertiles only in the IT region, but not in the NN and FS regions.

## DISCUSSION

In this community-based cohort study involving Korean men and women aged 65 years and older, we demonstrated that the femoral neck, and total hip BMD were significantly lower in women with lower normal TSH levels. In particular, we further analyzed the parameters for bone geometry of the proximal hip by using HSA; we observed deteriorated cortical bone parameters in the femur at all three regions (NN, IT, and FS) in women and at the IT in men in the lower TSH tertile as compared to the upper TSH tertile.

According to data from the fifth Korean National Health and Nutrition Examination survey (KNHANES) in 2010, the percentages of the population with osteoporosis among elderly men and women aged over 65 years were 15.2% and 57.7%, respectively [17]. In Korea, the prevalence of osteoporotic fractures is increasing with the rapid growth in the elderly population [18, 19]. Especially, since the rate of cortical bone loss becomes relatively accelerated after the age of 60 years, the prevalence of hip fractures is increasing rapidly in elderly individuals, particularly in women, placing a significant burden on public health services [20-24]. In addition, the risk of hip fractures increases with hyperthyroidism [25]. Abrahamsen et al. recently showed that factors increasing the risk of hip fractures included longer durations of thyrotoxicosis and low TSH levels at baseline [5]. Moreover, even in euthyroid subjects, the risk of hip fractures (hazard ratio [HR] 1.45, 95% CI 1.22-1.71, *p* < 0.001) and major osteoporotic fractures, including those of the hip, humerus, forearm, and spine (HR 1.32, 95% CI 1.19-1.46, *p* < 0.001), have been reported to significantly increase with each standard unit of TSH decrease [5].

**Table 3:** Odds ratios (ORs) for osteoporosis in the tertile groups of thyroid stimulating hormone (TSH) in euthyroid subjects

	Osteoporosis					
	Women			Men		
	OR	95% CI	*p* - value	OR	95% CI	*p* - value
1st tertile (TSH1)	1.86	(1.22 - 2.83)	< 0.01	1.72	(0.76 - 3.88)	0.19
2nd tertile (TSH2)	1.3	(0.86 - 1.97)	0.35	0.86	(0.35 - 2.09)	0.86
3rd tertile (TSH3)	Ref.			Ref.		

CI: confidence interval, Ref: reference group, TSH1: lower tertile, TSH2: middle tertile, TSH3: upper tertile. Multiple logistic regression analysis using osteoporosis as the dependent variable, adjusted by age, body mass index, free thyroxine, 25-hydroxyvitamin D, parathyroid hormone, smoking, drinking, the period of menopause (only for women).

Considering the relationship between thyroid function and hip fractures [2, 19, 26, 27], it can be concluded that thyroid hormones have a greater effect on cortical bone than on trabecular bone [28, 29]. Accordingly, as expected, the cortical geometry parameters, such as CSA and CoTh, were the lowest in subjects with lower normal TSH in the present study. Moreover, BR, a surrogate parameter of cortical instability, was the highest in the lower TSH tertile in euthyroid women, as compared to the upper TSH tertile, in all three regions (NN, IT, and FS) after adjustment for age, BMI, free thyroxine, 25(OH)D, PTH, smoking, drinking, and the period of menopause (only for women). Our findings of decreased proximal femoral BMD and deteriorated cortical bone geometry in this lower normal TSH range in women aged over 65 years may explain the recently reported association between the increased risk of hip fractures and the lower normal range of TSH in euthyroid subjects [9].

**Table 4:** Association between lower TSH tertile and bone geometry parameters in narrow neck, intertrochanter, and femur shaft.

Region	Geometry parameters	Women				Men		
		ß	SE	*p*-value		ß	SE	*p*-value
NN	CSA	-0.096	0.031	0.02		-0.062	0.056	0.26
	CoTh	-0.13	0.002	< 0.01		-0.067	0.004	0.24
	BR	0.111	0.319	0.01		0.105	0.434	0.08
IT	CSA	-0.113	0.06	< 0.01		-0.116	0.13	0.04
	CoTh	-0.14	0.006	< 0.01		-0.118	0.011	0.04
	BR	0.16	0.218	< 0.01		0.114	0.254	0.04
FS	CSA	-0.096	0.04	0.02		-0.04	0.078	0.47
	CoTh	-0.138	0.008	< 0.01		-0.032	0.014	0.58
	BR	0.142	0.075	< 0.01		0.075	0.084	0.22

NN: narrow neck, IT: intertrochanter, FS: femoral shaft, CSA: cross-sectional area, CoTh: cortical thickness, BR: buckling ratio. Multiple linear regression analysis performed with cortical geometry parameters of all three regions as dependent variables. Each multivariate regressions were done for each dependent variable, at a time, not simultaneously. Coefficients (ß) was shown by comparing the lower TSH tertile with the upper TSH tertile as a standard error (SE). Adjusted by age, BMI, free thyroxine, 25(OH)D, PTH, smoking, drinking, and the period of menopause (only for women) as covariates.

**Supplemental Table 1:** Bone geometry parameters according to the thyroid-stimulating hormone (TSH) tertile groups

	Women					Men				
	Total	TSH1	TSH2	TSH3	P	Total	TSH1	TSH2	TSH3	P
		n = 227	n = 227	n = 220	value		n = 112	n = 119	n = 112	value
TSH range		(0.36 - 1.17)	(1.18 - 1.93)	(1.94 - 5.50)			(0.36 - 1.05)	(1.06 - 1.67)	(1.68 - 5.50)	
Narrow neck										
CSA, cm ^2^	2.25 ± 0.35	2.20 ± 0.37†	2.24 ± 0.33	2.31 ± 0.36	0.01	2.91 ± 0.48	2.89 ± 0.49	2.90 ± 0.44	2.947 ± 0.52	0.67
CSMI, cm ^4^	1.95 ± 0.48	1.95 ± 0.52	1.95 ± 0.45	1.95 ± 0.47	0.99	3.29 ± 0.78	3.26 ± 0.73	3.29 ± 0.79	3.312 ± 0.83	0.89
Z, cm ^3^	1.07 ± 0.22	1.06 ± 0.23	1.08 ± 0.20	1.08 ± 0.23	0.61	1.62 ± 0.33	1.60 ± 0.31	1.63 ± 0.31	1.637 ± 0.36	0.72
CoTh, cm	0.14 ± 0.03	0.14 ± 0.03†	0.14 ± 0.02	0.15 ± 0.03	0.00	0.16 ± 0.03	0.16 ± 0.03	0.16 ± 0.03	0.165 ± 0.03	0.68
BR	13.38 ± 3.45	13.99 ± 3.77*†	13.15 ± 3.00	12.99 ± 3.47	0.01	12.96 ± 3.43	13.38 ± 4.41	12.84 ± 2.99	12.665 ± 2.67	0.34
Intertrochanter										
CSA, cm ^2^	3.78 ± 0.68	3.68 ± 0.69†	3.77 ± 0.64	3.89 ± 0.68	0.01	5.10 ± 1.09	4.97 ± 1.18	5.129 ± 1.10	5.19 ± 0.99	0.34
CSMI, cm ^4^	9.97 ± 2.59	9.79 ± 2.70	9.91 ± 2.51	10.23 ± 2.57	0.19	16.47 ± 6.04	15.82 ± 6.47	16.48 ± 5.68	17.10 ± 5.96	0.32
Z, cm ^3^	3.19 ± 0.74	3.11 ± 0.76†	3.17 ± 0.71	3.30 ± 0.73	0.02	4.79 ± 1.42	4.63 ± 1.53	4.81 ± 1.37	4.93 ± 1.35	0.32
CoTh, cm	0.31 ± 0.06	0.30 ± 0.07†	0.30 ± 0.06†	0.32 ± 0.06	<0.01	0.39 ± 0.09	0.38 ± 0.10	0.39 ± 0.08	0.40 ± 0.08	0.34
BR	10.62 ± 2.47	11.10 ± 2.70†	10.64 ± 2.21	10.12 ± 2.38	<0.01	9.13 ± 2.13	9.40 ± 2.47	9.05 ± 2.04	8.95 ± 1.82	0.30
Femur shaft										
CSA, cm ^2^	3.59 ± 0.47	3.51 ± 0.47†	3.59 ± 0.46	3.66 ± 0.46	<0.01	4.73 ± 0.66	4.71 ± 0.69	4.72 ± 0.63	4.77 ± 0.66	0.76
CSMI, cm ^4^	3.15 ± 0.61	3.13 ± 0.61	3.19 ± 0.61	3.14 ± 0.61	0.55	4.70 ± 0.95	4.68 ± 0.86	4.68 ± 0.97	4.73 ± 1.02	0.90
Z, cm ^3^	2.07 ± 0.30	2.04 ± 0.30	2.08 ± 0.30	2.08 ± 0.30	0.41	2.85 ± 0.43	2.84 ± 0.40	2.85 ± 0.43	2.86 ± 0.46	0.93
CoTh, cm	0.47 ± 0.09	0.46 ± 0.09†	0.47 ± 0.08†	0.49 ± 0.09	<0.01	0.60 ± 0.11	0.60 ± 0.13	0.60 ± 0.09	0.60 ± 0.11	0.80
BR	3.38 ± 0.81	3.50 ± 0.84†	3.42 ± 0.79†	3.21 ± 0.76	<0.01	2.85 ± 0.65	2.91 ± 0.77	2.83 ± 0.57	2.81 ± 0.61	0.51

Values presented as the mean ± standard deviation.
TSH1: lower tertile, TSH2: middle tertile, TSH3: upper tertile, CSA: cross-sectional area, CSMI: cross sectional moment of inertia, Z: section modulus, CoTh: cortical thickness, BR: buckling ratio.
Bonferroni’s post hoc analysis: *p <0.05 vs. TSH2, †*p* <0.05 vs. TSH3. *P* values were calculated by ANOVA.

In the present study, the lumbar spine BMD tended to be lower as the TSH level decreased, consistent with previous results [30]. However, the prevalence of vertebral fractures, detected by lateral X-ray, was not significantly associated with the lower TSH tertile group compared to the upper TSH group in both men and women. In contrast to our study, Mazziotti et al. showed that the vertebral fracture prevalence was the highest in the lower tertile of TSH values in euthyroid postmenopausal women [31]. One possible reason for the distinct findings compared to our results could be differences in the age of the subjects. In the previous study, the age of the subjects, which is an important factor affecting the risk of osteoporosis and spinal fractures, in the first tertile of serum TSH was significantly older (mean age: 71 years) than that in the third tertile (mean age: 65 years). Moreover, early postmenopausal women were also included in the analysis, and the presence of rapid trabecular bone loss due to estrogen deficiency following menopause could hence not be excluded. As estrogen is an important factor affecting the cortical and trabecular bone compartment in the perimenopausal period, estrogen may counteract the negative skeletal effects of thyroid hormone, and the relationship between thyroid status and bone parameters could thus not be evaluated fully in the relatively young subjects.

Regarding the effect of thyroid status on bone in men, Roef et al. showed that the thyroid hormone status affected bone mass maximally at the bone building age [29]; however, no correlation between BMD and TSH was noted in men aged over 50 years, consistent with our findings [9, 32]. Although the BMD was not related with the TSH level, our result showed that the geometry parameters, including bone strength parameters (CSMI, Z), in the IT region were associated with the TSH tertile level in men. The different effects of TSH on endocortical resorption in men and women could be a possible reason for the observed differences between the sexes. Moreover, the lower number of men compared to women in the present study may be another reason for the weaker association in men.

Previous data have demonstrated that high physiological levels of fT4 are related to BMD in women [8, 33], while T3 has been reported to directly promote osteoblastic proliferation, differentiation, and apoptosis [34]. In addition, TSH may also have direct effects on bone remodeling in experimental animals [19] as well as in humans [35]. Abe et al. showed evidence for the direct effects of TSH on bone, revealing high-turnover osteoporosis in *Tshr^-/-^* mice with normal circulating T4 and T3 levels, along with regulation of osteoclast formation and osteoblast differentiation by TSH [19]. Further, Mazziotti et al. showed the inhibitory effect of TSH on bone resorption in vivo by recombinant human TSH [35]. In our study, the tertiles of fT4 were not found to be associated with BMD or any of the bone geometry parameters in men, while the fT4 tertiles were only associated with CSA and CoTh of the FS in women (data not shown).

This study has certain limitations. First, this was a cross-sectional study. However, this cohort is scheduled to be followed-up for 10 years. During this follow-up, the occurrence of fractures and the measurement parameters for longitudinal changes in BMD and bone geometry will be recorded. Second, the DXA technique is limited by its imperfect analysis of the cortical bone compartment. The HSA system assumes that the cross-section of the femoral regions is a circle or eclipse, although, in reality, there are regional differences in the ratio of the cortical compartment to the trabecular compartment around the NN [36, 37]. High-resolution peripheral quantitative computed tomography may be useful for analyzing the cortical bone compartments in the extremities [11]; however, this modality cannot analyze the proximal hip structure. Nonetheless, as DXA is the most widely used method worldwide, HSA-derived geometry parameters can be relatively easily analyzed as important markers for predicting hip fractures.

Despite its limitations, the current study also has important strengths. To the best of our knowledge, this is the first study to analyze the detrimental effects of low-normal TSH levels on the hip geometry parameters of the NN, IT, and FS in both men and women. We demonstrated that lower normal TSH levels could be considered a predictive factor for weakening femoral bone strength in elderly women, indicating that more careful assessment for osteoporosis and fracture risk is needed in this population.

In conclusion, our study suggested that lower TSH levels, even within the normal range, negatively affect the BMD of the hip as well as the proximal hip geometry, providing a plausible explanation for the increased risk of hip fractures observed in elderly euthyroid women. Acknowledgments
